# Pneumatosis intestinalis induced by osimertinib in a patient with lung adenocarcinoma harbouring epidermal growth factor receptor gene mutation with simultaneously detected exon 19 deletion and T790 M point mutation: a case report

**DOI:** 10.1186/s12885-019-5399-5

**Published:** 2019-02-28

**Authors:** Yuki Nukii, Atsushi Miyamoto, Sayaka Mochizuki, Shuhei Moriguchi, Yui Takahashi, Kazumasa Ogawa, Kyoko Murase, Shigeo Hanada, Hironori Uruga, Hisashi Takaya, Nasa Morokawa, Kazuma Kishi

**Affiliations:** 1 Department of Respiratory Medicine, Toranomon Hospital (Branch), 1-3-1 Kajigaya Takatsu-ku, Kawasaki-shi, Kanagawa 213-8587 Japan; 2 Department of Respiratory Medicine, Respiratory Center, Toranomon Hospital, 2-2-2 Toranomon Minato-ku, Tokyo, 105-8470 Japan

**Keywords:** Receptor, epidermal growth factor, Protein kinase inhibitors, Lung neoplasms, Osimertinib, Pneumatosis intestinalis

## Abstract

**Background:**

Pneumatosis intestinalis is a rare adverse event that occurs in patients with lung cancer, especially those undergoing treatment with epidermal growth factor receptor tyrosine kinase inhibitors (EGFR-TKI). Osimertinib is the most recently approved EGFR-TKI, and its usage is increasing in clinical practice for lung cancer patients who have mutations in the *EGFR* gene.

**Case presentation:**

A 74-year-old woman with clinical stage IV (T2aN2M1b) lung adenocarcinoma was determined to have *EGFR* gene mutations, namely a deletion in exon 19 and a point mutation (T790 M) in exon 20. Osimertinib was started as seventh-line therapy. Follow-up computed tomography on the 97th day after osimertinib administration incidentally demonstrated intra-mural air in the transverse colon, as well as intrahepatic portal vein gas. Pneumatosis intestinalis and portal vein gas improved by fasting and temporary interruption of osimertinib. Osimertinib was then restarted and continued without recurrence of pneumatosis intestinalis. Overall, following progression-free survival of 12.2 months, with an overall duration of administration of 19.4 months (581 days), osimertinib was continued during beyond-progressive disease status, until a few days before the patient died of lung cancer.

**Conclusions:**

Pneumatosis intestinalis should be noted as an important adverse event that can occur with administration of osimertinib; thus far, such an event has never been reported. This was a valuable case in which osimertinib was successfully restarted after complete recovery from pneumatosis intestinalis, such that further extended administration of osimertinib was achieved.

## Background

Pneumatosis intestinalis is a disease in which air-containing cysts form within the submucosa or serosa of the intestinal tract [1]. Although the detailed pathogenesis or aetiology of intra-mural gas formation remains unknown, several mechanisms have been proposed, such as a bacterial theory and a mechanical theory [2, 3]. Overall, the development of pneumatosis intestinalis is believed to be multifactorial in individual patients, because the underlying diseases or medical conditions in each reported case have been considerably different [2]. Pneumatosis intestinalis can be secondary to several conditions such as digestive tract obstruction, ischemic bowel disease, inflammatory bowel disease, autoimmune disease, infectious enteritis, intestinal tumour, and trauma [3]. The severity varies from benign to life-threatening disease [4]. Pneumatosis intestinalis can also be secondary to some drugs, such as molecular targeted therapy agents [5], corticosteroids [4], and alpha-glucosidase inhibitors [6].

Pneumatosis intestinalis occurring during treatment for lung cancer has been reported previously, especially in patients receiving epidermal growth factor receptor (EGFR) tyrosine kinase inhibitors (TKIs), such as gefitinib and erlotinib [7–15]. In addition, pneumatosis intestinalis has been reported in patients treated with cytotoxic agents, such as amrubicin [16], S-1 plus oral leucovorin combination [17], carboplatin plus irinotecan combination [18], and cisplatin + irinotecan combination [19]. Osimertinib is the most recently approved third-generation EGFR-TKI, which can be effective in patients with advanced-stage lung adenocarcinoma harbouring an *EGFR* gene mutation and an acquired drug-resistant mutation, such as the exon 20 T790 M point mutation [20]. Furthermore, clinical benefits for use of osimertinib as first-line treatment in patients harbouring so-called common *EGFR* gene mutations (the exon 21 L858R point mutation and the exon 19 deletions) were proven in the FLAURA study [21]; subsequently, an increasing number of patients with anticancer therapy naïve, *EGFR* gene mutation positive advanced non-small cell lung cancer have received osimertinib. Here, we report a case of osimertinib-induced pneumatosis intestinalis.

## Case presentation

**Fig. 1:**
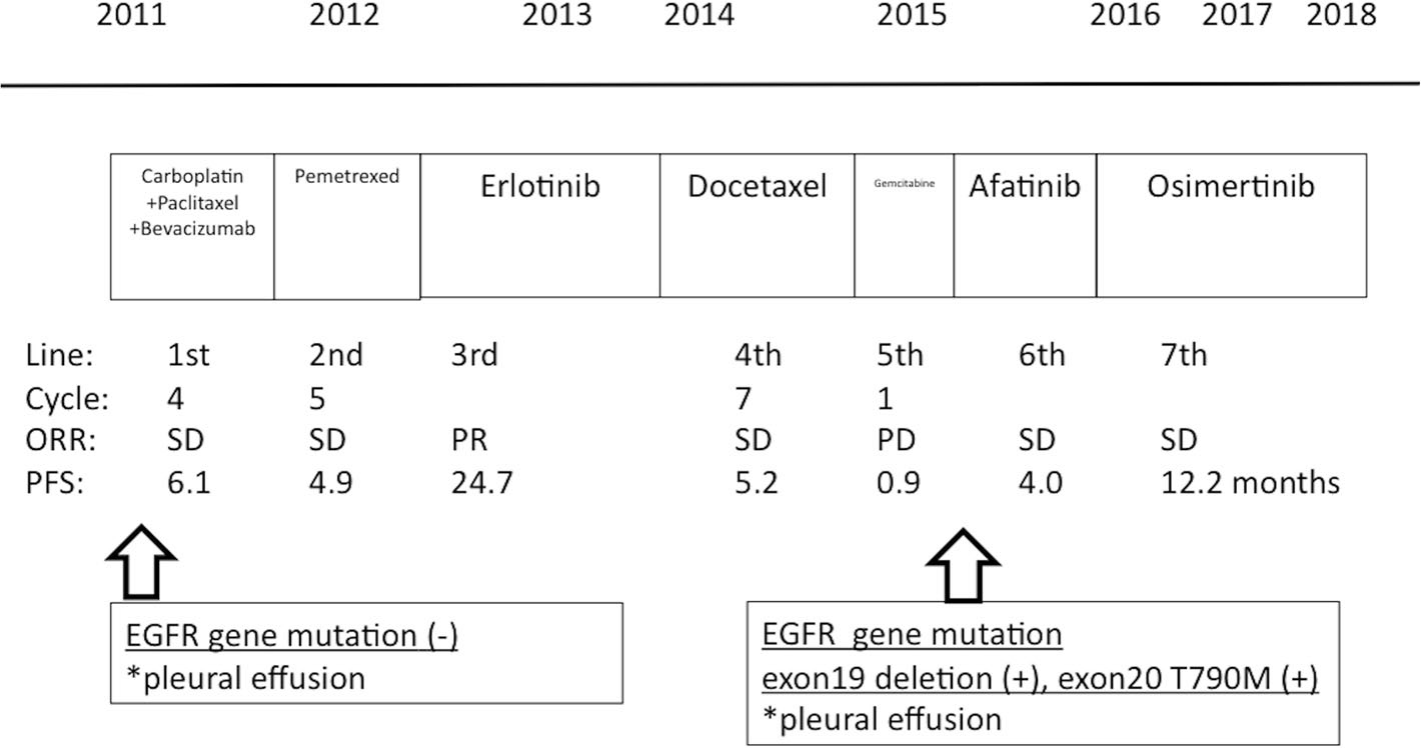
Timeline of anticancer treatments. Progression-free survival and best objective response of each regimen are summarized. Abbreviations: ORR: objective response rate, PD: progressive disease, PFS: progression-free survival, PR: partial response, SD: stable disease

A 69-year-old Japanese woman who had never smoked was initially diagnosed with clinical stage IV (T2aN2M1b in 7th edition) lung adenocarcinoma with pleural and bone metastasis. She had no history of chronic obstructive pulmonary disease, diabetes mellitus, or any colonic diseases (such as constipation). At the initial diagnosis, no *EGFR* gene mutation was detected in malignant pleural effusion by real-time polymerase chain reaction (PCR). A combination regimen with carboplatin, paclitaxel, and bevacizumab was started as the first-line treatment (Fig. 1). Next, pemetrexed, erlotinib, and docetaxel were administered as second-, third-, and fourth-line treatments, respectively. Each regimen was changed because of disease progression. Lung cancer progressed with increased pleural effusion after one cycle with gemcitabine (fifth-line treatment). Therefore, *EGFR* gene mutation was studied in pleural effusion, using the PCR fragment analysis/PCR clamp method, because the progression-free survival (PFS) of erlotinib was 24.7 months. Two *EGFR* gene mutations were detected, namely a deletion in exon 19 and a T790 M point mutation in exon 20. Based on the genetic results, afatinib was started as the sixth-line treatment, as recommended in the LUX-Lung-4 study [22]. Osimertinib was not an option because it was not yet approved at that time. The PFS of afatinib was 4.0 months. Treatment with afatinib was continued for 15.3 months (458 days) until osimertinib was approved.

**Fig. 2:**
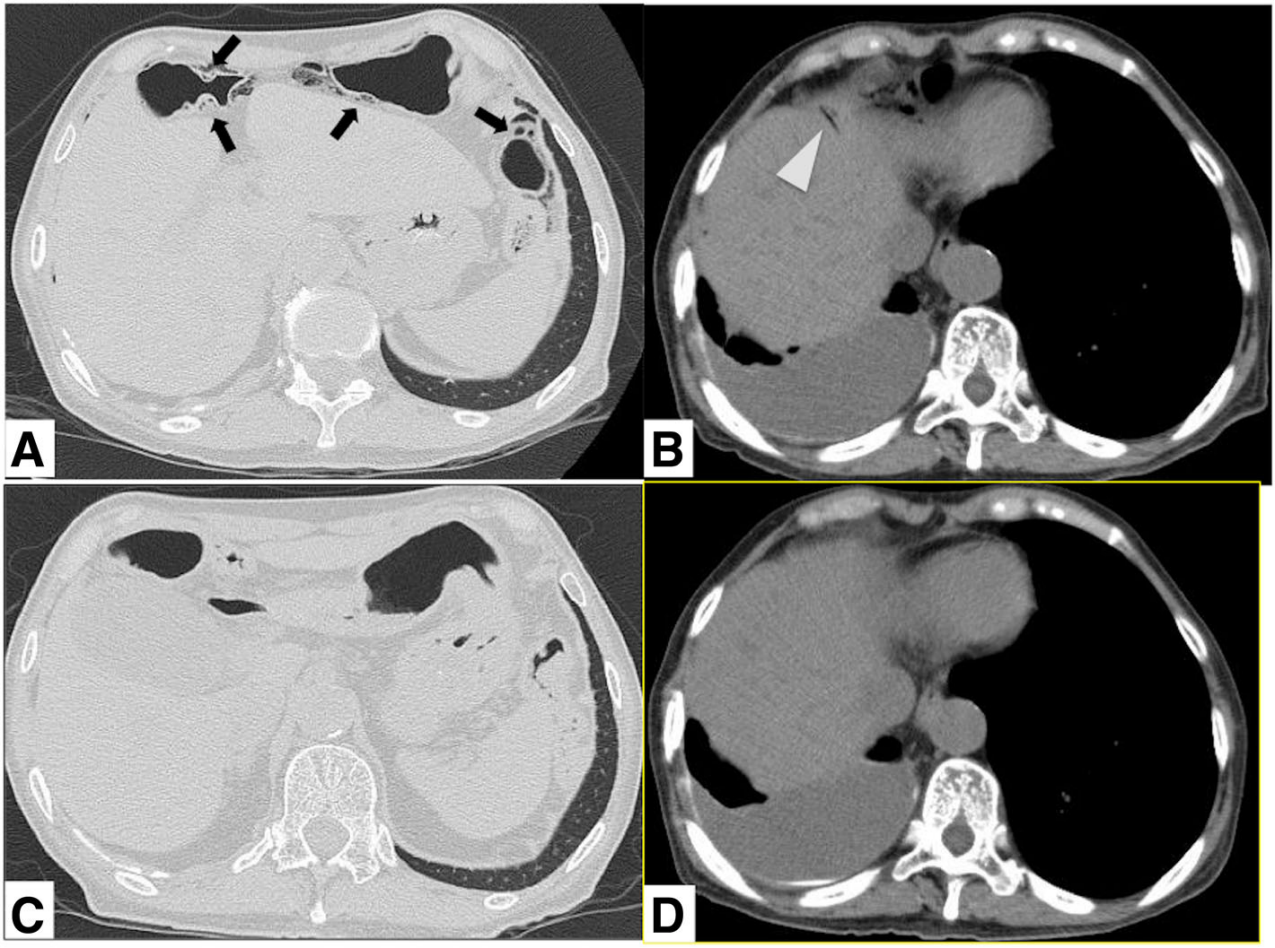
Abdominal computed tomography. **a** Intra-mural gas (arrows) in the wall of the transverse colon. **b** Intra-hepatic portal vein gas (arrowhead) in the peripheral area of the liver. **c**, **d** After treatment, both of these findings were improved

**Fig. 3:**
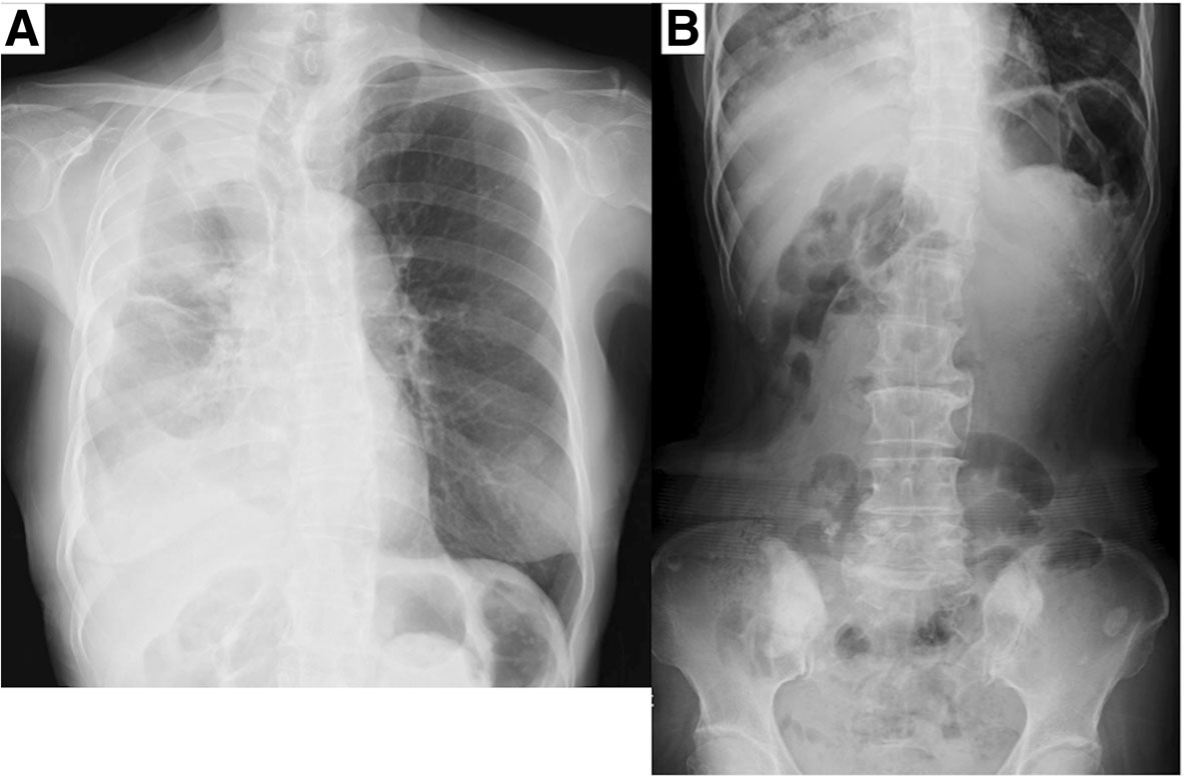
Chest and abdominal X-ray. **a** Right-sided pleural effusion due to lung cancer. No evidence of free air in the abdominal cavity. **b** No evidence of overt gas along the wall of the intestinal tract

Osimertinib (80 mg/day) was started as the seventh-line treatment at her age of 74, when the patient had a body mass index of 16.2 kg/cm^2^ and a performance status of 1. The adverse events, cutaneous pruritus and stomatitis, were graded with Common Terminology Criteria for Adverse Events (CTCAE, ver 4.0) as grade 1. However, there was gradual improvement in the shoulder pain that had resulted from bone metastasis, and oral administration of oxycodone was successfully stopped on the 87th day after osimertinib was started. The best response of osimertinib was stable disease. In follow-up computed tomography (CT) at day 97 after treatment with osimertinib, intra-mural air in the transverse colon and intra-hepatic portal vein gas were incidentally observed. Intra-mural air in the bowel intestine was considered to be pneumatosis intestinalis. However, no evidence of perforation was observed because free air was absent from the abdominal cavity (Figs. 2a, b and 3). Progression of lung cancer was not recognized.

Because of the close association between pneumatosis intestinalis and EGFR-TKI usage [5], pneumatosis intestinalis was considered to have been induced by osimertinib, which had been most recently administered. There were no subjective symptoms, such as abdominal pain. Her white blood cell count was 3500/μL (normal range: 3400-9200/μL), serum C-reactive protein was 0.0 mg/dL (normal range: 0.0–0.3 mg/dL), and creatinine kinase (CK) was 48 IU/L (normal range: 62–287 IU/L). Therefore, there were no findings to indicate inflammatory response, such as infection or intestinal necrosis.

We put the patient on fasting conservatively and interrupted osimertinib on the first day of fasting, monitoring her condition carefully. She received neither antibiotics, nor an oxygen supply. After improvement of pneumatosis intestinalis and intra-hepatic portal vein gas on abdominal CT taken on day 7 (Fig. 2c, d), we resumed meals orally on day 7 and osimertinib (80 mg/day) on day 10.

It had been possible to continue osimertinib successfully. With a PFS of 12.2 months, osimertinib was continued with beyond-progressive disease status. Our patient was administered osimertinib until a few days before she died of lung cancer. The total duration of osimertinib administration was 19.4 months (581 days) (16.1 months [484 days] since complete recovery from pneumatosis intestinalis). Her overall survival was 79.3 months.

## Discussion and conclusions

Here, we report a case of pneumatosis intestinalis and intra-hepatic portal vein gas that occurred during administration of osimertinib to a patient with advanced-stage lung adenocarcinoma harbouring *EGFR* gene mutations (a deletion in exon 19 and a T790 M point mutation in exon 20). Pneumatosis intestinalis was resolved by conservative treatment and temporary interruption of osimertinib. Even after osimertinib had been restarted, no recurrence was observed during the remaining follow-up period.

Pneumatosis intestinalis is a disease in which air-containing cysts form within the submucosa or serosa of the intestinal tract [1]. Some theories that air intrudes to the intestinal wall have been proposed [3, 23]: i) according to the mechanical theory, some mechanical triggers, such as drugs or immune dysfunction, may lead to disruption of the submucosa, fragility of integration of mucosal epithelia, intestinal wall injury, and increased intestinal pressure; ii) according to the bacterial theory, the bacilli invading the submucosa may promote mucosal breaks, increase submucosal permeability, and contribute to the production of gas within the submucosa; iii) according to the chemical/nutritional deficiency theory, some medicines such as alpha-glucosidase inhibitors, or malnutrition, may change the bacterial flora in the bowel intestine, leading to an increase in gas production; and iv) according to the pulmonary theory, the rupture of alveolar structures may increase the entry of air into the mesenteric vessels via the pneumomediastinum. Although the details of the mechanisms remain unknown, multifactorial triggers may cause pneumatosis intestinalis.

The detailed mechanism by which EGFR-TKIs cause pneumatosis intestinalis is not fully understood. However, a previous report has suggested that EGFR-TKIs could lead to pneumatosis intestinalis through the following process: i) the basic fibroblast growth factor, one of the growth factors expressed in the gastrointestinal tract, is involved in maintaining the gastrointestinal mucosa (epithelium) in a murine model [24]; and ii) gefitinib damages the gastrointestinal mucosa and impairs its defence and repair function [9]. These pharmacological actions of EGFR-TKIs may ultimately cause pneumatosis intestinalis. EGFR-TKIs, including osimertinib, may share a similar mechanism that impairs the turnover of intestinal epithelial cells.

Our patient had none of the other risk factors for pneumatosis intestinalis that have been reported previously, such as diabetes mellitus, steroid or narcotic use, underlying colonic disease, and pre-existing acute colonic infection. Although she had undergone long-term administration of multiple potentially causative agents for pneumatosis intestinalis, including other EGFR-TKIs and bevacizumab (Fig. 1), her first episode of pneumatosis intestinalis did not occur until osimertinib treatment was initiated. Because oxycodone was successfully stopped only 10 days before the diagnosis of pneumatosis intestinalis, the patient’s risk of pneumatosis intestinalis had been reduced at the time that pneumatosis intestinalis was diagnosed.

Mild disease is usually asymptomatic and pneumatosis intestinalis is detected by routine CT study incidentally with median duration until detection with less than 3 months [5]. Mild disease usually improves with conservative treatment and withdrawal of EGFR-TKI treatment. The typical symptoms of pneumatosis intestinalis are abdominal pain, diarrhoea, distention, nausea/vomiting, bloody/mucous stool, and constipation [5]. These symptoms may be observed in severe disease, especially when both intestinal perforation and peritonitis occur. These manifestations may lead the patients to emergency surgery [2, 25]. The current case has neither overt symptoms nor any laboratory findings suggestive of intestinal necrosis or severe disease.

Radiologically, the characteristic findings have been investigated. Abdominal CT features suggestive of severe life-threatening disease have been reported as bowel wall thickening, mesenteric stranding, ascites, bowel dilatation, location confined to small bowel, and portomesenteric venous gas [4]. In the current case, none of the aforementioned CT features suggestive of life-threatening disease were observed (Fig. 2a, b).

Intra-hepatic portal vein gas is probably air that intrudes into the portal vein via the intestinal tract [26]. It is commonly observed in combination with pneumatosis intestinalis [9]. Furthermore, its identification is usually, but not always, helpful to distinguish severe from mild disease, and it is strongly associated with mesenteric ischemia [1]. In the current case, the extent of intra-hepatic portal vein gas was very mild (Fig. 2b). This may be the reason why the presence of intra-hepatic portal vein gas was not associated with severe mesenteric ischemia in this case.

There are reports of nine cases of patients with pneumatosis intestinalis caused by EGFR-TKI usage (Table 1) [7–15]. Gefitinib and erlotinib were used in six and three patients, respectively. Among all but one, conservative observation and withdrawal of EGFR-TKIs were chosen as the major treatments (Table 1). Iwasaku et al. [8], Otsubo et al. [11], and Maeda et al. [12] reported patients who were re-administered EGFR-TKIs after the symptom of mild abdominal pain improved. Yamamoto et al. [14] reported another case of pneumatosis intestinalis that was resolved without interruption of EGFR-TKI treatment. In that case, none of the aforementioned clinical and radiological findings suggestive of life-threatening disease were observed, and the extent of the involved areas was limited in our case. All prior cases have occurred in Asian countries, primarily Japan [7–15]. It is well-known that there is an ethnic difference in the prevalence of *EGFR* positive lung cancer between Western and Asian populations [27, 28]. The prevalence of pneumatosis intestinalis may correspond to that of *EGFR* gene mutation positive lung cancer. Upon review of prior case reports with regard to Asian patients, no risk factors other than those previously reported to cause pneumatosis intestinalis were found.

**Table 1:** Reported cases of pneumatosis intestinalis in patients with lung cancer who received treatment with EGFR-TKIs

Drug	First author	Publication year	Age/sex	Treatment	Symptoms	Complication	Outcome	Restart of EGFR-TKI treatment	Recurrence
Gefitinib	Higashino (7)	2010	67/F	Observation/withdrawal	Mild abdominal pain	Intra-peritoneal free air	Improved	ND	–
Gefitinib	Iwasaku (8)	2012	82/F	Observation/withdrawal	Mild abdominal pain	None	Improved	Yes	Yes
Gefitinib	Lee (9)	2012	66/F	Observation/withdrawal	Vomiting, diarrhea, and abdominal distension	Intra-hepatic portal veinous gas with suggestive of liver infarction	Improved	No	–
Gefitinib	Wakabayashi (10)	2012	83/M	Surgery/ withdrawal	Fever, vomiting, diarrhea, and severe abdominal pain	Intra-peritoneal free air, remarkable elevation of inflammatory parameters	Improved	ND	–
Gefitinib	Otsubo (11)	2015	71/M	Observation/withdrawal	Vomiting, diarrhea, and abdominal pain	Intra-peritoneal free air	Improved	Yes	No
Gefitinib	Maeda (12)	2016	80/F	Observation/withdrawal	Anorexia, constipation, and abdominal distension	Intra-peritoneal free air, mild elevation of inflammatory parameters	Improved	Yes	Yes
Erlotinib + bevacizumab	Tsukita (13)	2014	69/M	Observation/withdrawal	No symptoms	Intra-peritoneal free air	Improved	ND	–
Erlotinib + pemetrexed	Yamamoto (14)	2014	70/F	Observation, continuation of erlotinib	Vomiting, anorexia, diarrhea, and abdominal pain	None	Improved	–	–
Erlotinib + bevacizumab	Saito (15)	2016	73/F	Observation/withdrawal	Anorexia and fatigue	Intra-peritoneal free air	Improved	No	–
Osimertinib	Current case	2018	73/F	Observation/withdrawal	No symptoms	Intra-hepatic portal vein gas	Improved	Yes	No

*Abbreviations: EGFR-TKI* epidermal growth factor receptor tyrosine kinase inhibitor, *F* female, *M* male, *ND* not described

Because our patient had already experienced six regimens of anticancer therapy, osimertinib was considered to be the singular option that had the potential to stabilize the progression of lung cancer. The restart and continuation of osimertinib under close observation was a meaningful approach, which resulting in the patient maintaining PFS for 19.4 months. Our patient had essentially no underlying risk factors, and the change of the EGFR-TKI from afatinib to osimertinib was the only way in which her condition was altered during the time that pneumatosis intestinalis developed. The absence of recurrence after restarting osimertinib might be explained by the use of dietotherapy that focused on ease of digestion and the avoidance of factors that could have exacerbated pneumatosis intestinalis, such as constipation.

Pneumatosis intestinalis is a rare adverse event that can occur in patients with lung cancer treated with osimertinib. In this case, it was a valuable and successful option to restarted osimertinib after the patient had completely recovered from pneumatosis intestinalis without deterioration, resulting in extended administration of osimertinib.

## Abbreviations

CK: Creatinine kinase
CT: Computed tomography
CTCAE: Common terminology criteria for adverse event
EGFR: Epidermal growth factor
ORR: Objective response rate
PCR: Polymerase chain reaction
PD: Progressive disease
PFS: Progression-free survival
PR: Partial response
SD: Stable disease
TKI: Tyrosine kinase inhibitor
